# A Novel Bayesian Seamless Phase I/II Design

**DOI:** 10.1371/journal.pone.0073060

**Published:** 2013-09-04

**Authors:** Haitao Pan, Ping Huang, Zuoren Wang, Ling Wang, Chanjuan Li, Jielai Xia

**Affiliations:** 1 Department of Health Statistics, Fourth Military Medical University, Xi’an, China; 2 School of Statistics, Xi’an University of Finance and Economics, Xi’an, China; 3 Department of Clinical Laboratory, School of Stomatology, Fourth Military Medical University, Xi’an, China; New Jersey Institute of Technology, United States of America

## Abstract

This paper proposes a novel bayesian phase I/II design featuring using a hybrid mTPI method in phase I for targeting the MTD level and a randomization allocation schema for adaptively assigning patients to desirable doses in phase II. The mechanism of simultaneously escalating dose in phase I and expanding promising doses to phase II is inherited from a design proposed in literature. Extensive simulation studies indicate that our proposed design can vastly save sample size and efficiently assign more patients to optimal dose when compared to two competing designs.

## Introduction

Though some designs, under the name of phase I/II, have been proposed recent years. Most of them target the maximum tolerated dose(MTD) level(MTD refers to the highest dose that will produce the desired effect without unacceptable toxicity.), which should, as the authors believe, bear a more appropriate name: phase I/II dose finding design([1], [2], [3], [4], [5], [6]). Based on our knowledge, only several proposed designs could be titled as “true” seamless phase I/II design, among them, there are XJT design proposed by Xie etc.([7]) and three-stage design proposed by Pan etc ([8]),which are currently available. While both of them show good performances, the three-stage design outperforms the XJT design. However, at the phase I stage, the three stage design still failed to use more flexible adaptive design, like CRM or mTPI designs(both are model-based Bayesian adaptive phase I designs). In this paper, we equip the three-stage design with the advanced mTPI design. More steps have been made in this paper: we adopt the hybrid mTPI in phase I and conduct extensive simulations to compare their performances to draw final conclusion. The major reason that we select the mTPI instead of CRM design to improve the efficiency in phase I of the three-stage design is that it has been proven to have the similar statistical performances to the CRM design yet simple to use([9], [10], [11]). We are aware that none of this kind of studies that equip the mTPI design with the integration of the phase I and II processes have been explored in the literature.

We briefly introduce the paradigm of the three-stage design as follows. The design’s three stages refer to phase I, phase IIa, and IIb, respectively. The design features integration of the processes of dose escalation and dose expansion. Dose escalation is guided by the 3+3 approach, which is a classical design and has been considered as the gold standard design in phase I trials([10]). Once a current administered dose is escalated and a new dose is opened for toxicity study, this current dose is expanded to phase IIa(stage 2) for preliminary research. The efficacy information is updated by a beta-binomial model. Stage 2 requires two interim analyses: a futility rule which determines when the current dose should be dropped out from the study and a graduation rule which informs whether the current dose should be graduated to phase IIb(stage 3) or not. In stage 3, an adaptive randomization procedure is implemented to assign the treated patients to desirable dose levels. Readers refer to([8]) for details.

This paper is organized as follows. Section 2 covers the scheme of mTPI design and its hybrid version. In Section 3, the general design structure is introduced in depth. Section 4 elaborates on the extensive simulation studies. Finally, Section 5 is devoted to discussions and conclusions.

## Phase I mTPI Design and its Hybrid Version

Firstly, we will introduce mTPI designs; and then the hybrid mTPI versions will be described.

### mTPI Design

The dose-finding rules for the mTPI method involve two major steps. In the first step, one introduces an equivalence interval (EI), which leads to three toxicity probability intervals that partition the probability space (0,1) into three intervals, corresponding to three conditions, namely, under-dosing, proper dosing, and over dosing, respectively. Building upon the EI, the mTPI method computes the unit probability mass (UPM, which is defined as the ratio of the probability the interval and the length of the interval) for the three toxicity probability intervals, and sets up a decision-theoretic framework to guide dose escalation decision on a Bayes rule basis.

Specifically, define 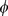 to be the target toxicity probability of the MTD (e.g, 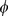 = 0.25). The goal of phase I clinical trials is to find the highest dose with a toxicity probability closest to 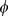. Let 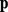 = 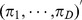 denote the toxicity probabilities for dose j = 1,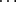,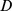, where 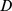 is the total number of candidate doses in the trial. The observed data include the 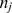 patients treated at dose j and the corresponding 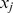 experiencing toxicity. The likelihood function is a product of binomial densities,
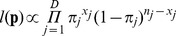
(1)


The mTPI design assumes independence among dose responses and proposes to use models with vague priors for 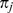 so that the shape of the resulting posterior distributions will be decided mainly by the shape of the likelihood based on the observed data. In this design, we set priors of 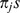 as Beta(1,1), with Beta density proportional to 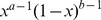. Combined with the likelihood in (1), the posterior of 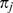 follows independent 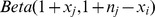, for 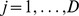. Evidently, when strong prior information on the toxicity of the candidate doses are available, informative beta priors can replace the vague priors.

Assume dose 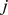 is currently used to treat patients. To apply mTPI, one simply calculates the three UPMs for under-, proper-, and over-dosing intervals, given by
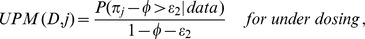


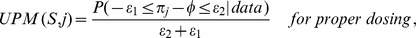


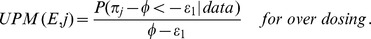



A dose-assignment rule 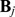 based on these three UPMs chooses the decision with the largest UPM, that is,
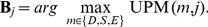
(2)


The mTPI design imposes an extra safety rule which restricts escalation to toxic doses that have been previously used. Introduing a random variable 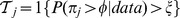, where 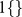 is the indicator function and 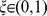 is a cutoff value (e.g.,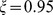), mTPI incorporates 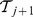 into the proposed dose-assignment rule 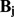. Let 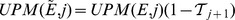 and define the new dose-assignment rule with this toxicity exclusion to be 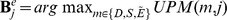. When 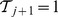, dose 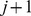 is considered highly toxic and the UPM associated with escalation equals 0. Therefore, escalation will never be chosen for dose finding. We recommend readers to refer to (Ji et al., 2010) [11] for details.

### Hybrid mTPI Design

The hybrid version of CRM design is advanced by Yuan & Yin ([12]). They have demonstrated that the hybrid CRM design outperforms the 3+3 and CRM designs. We borrow their essential idea here to construct a hybrid mTPI version, which inherits the robustness of bayesian hybrid dose-finding method. Specifically, in phase I, if the current observed information is informative enough for us to know whether this dose is below or above the MTD, we could make the relevant dose assignment decision (e.g.,either to escalate or deescalate or stay at the current dose) instantly without using advanced adaptive phase I design. If the information observed at the current dose is insufficient to make a definite decision, we will adopt the mTPI design so that borrowing strength across all the doses under study to guide proper dose assignment is feasible. The following is the detailed introduction of the hybrid mTPI design.

Suppose 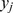 out of 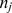 patients have experienced toxicity with the dose level 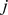. To evaluate the distance between the toxicity probability of dose level 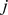 and the target toxicity probability of the MTD 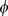, the following hypotheses are introduced:
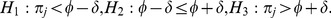
(3)where 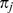 is the toxicity probability of dose level 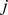, and 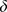 is the tolerable margin prespecified by physicians. The hypotheses 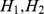 and 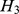 represent the situation in which dose level 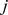 is below, approximately equal to, and above the MTD, respectively. We set up 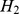 as an interval hypothesis 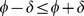 rather than a traditional point hypothesis 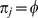 in that in clinical practice, as long as the toxicity probability of a dose is adequately close to 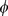, this dose can be chosen as the MTD. For example, with 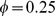 and 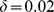, a dose with a toxicity probability within 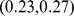 would be accepted as the MTD.

Given the data observed at dose level 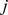, 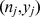, we derive the evidence of supporting each hypothesis by calculating their posterior probabilities. We assign the toxic probabilities 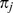 as a Beta(1,1) prior distribution under each hypothesis:
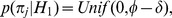


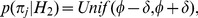
(4)

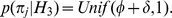



It then follows that the marginal distribution of 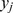 under 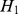 is given by
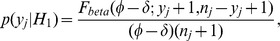
where 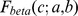 is the cumulative distribution function of a beta distribution with the shape and scale parameters 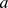 and 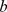 at the value 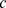. Similarly, the marginal distributions of 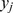 under 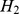 and 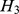 are given by



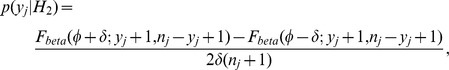
and



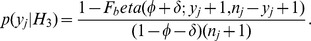
respectively. Therefore, at dose level 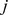, the posterior probability of 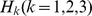 is given by



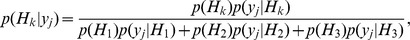
which is equivalently to the following:
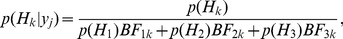
(5)where 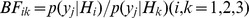, is the Bayes factor of 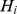 against 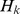.

To determine the magnitude of the evidence in favor of each hypothesis,more specifically, Jeffreys([14]) suggested interpreting the Bayes factor in the unit of 1/2 on the 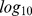 scale: if 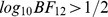, this indicates that the data contain substantial evidence in favor of 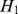 against 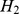; if 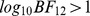, such evidence is strong in the data; and if 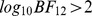, then the evidence appears to be decisive. In our case, if 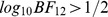 and 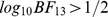, or equivalently 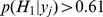, there is substantial evidence in favor of 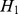 against both 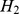 and 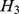, suggesting that dose level 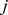 is far below the MTD. As a result, we should directly escalate the dose to level 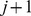, without the need to borrow any information from other doses. Similarly, if 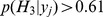, we should de-escalate the dose to level 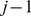 as there is substantial evidence indicating that dose level 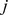 is far above the MTD. Finally, if 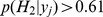, there is substantial evidence that dose level 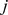 is close to the MTD, the next dose should then stay at the same level.

When none of the posterior probabilities of the hypotheses is greater than 0.61, that is, 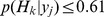 for all 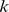, then that’s not informative enough at dose level 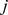 to support any action. As a consequence, we invoke the mTPI approach to pool the information together from all the dose levels to guide the dose assignment for new patients. In other words, if the toxicity information at the currently administered dose is strong enough, we draw the decision upon the Bayes factors obtained in (5); otherwise we resort to the model-based approach to borrow information across different dose levels.

## Design

We replace the 3+3 method in the three-stage design with the above hybrid mTPI approach. After the completion of phase I stage, the adaptive randomization approach by Yuan & Yin([15]) is adopted to effectively assign patients to the ideal dose level. Our design uses the beta-binomial model for efficacy responses. Let 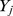 denotes the number of responses among the 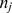 patients treated with dose arm 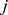 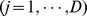 and 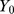 is the number of responses among the 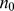 patients treated with placebo arm. Let 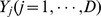 and 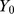 be independent random variables following the binomial distribution, Bin(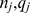), and Bin(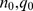), respectively. The joint likelihood function for all doses can be written as 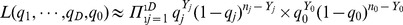. In our design, the response rates 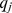 and 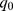 are assumed to be independent and identically distributed with Beta(0.5,0.5), where Beta(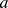,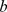) denotes a beta distribution, and its density is proportional to 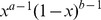. Based on the Bayesian theory, the posterior distribution of 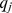 is Beta(0.5+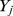, 0.5+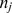
−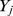) and the posterior distribution of 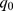 is Beta(0.5+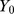, 0.5+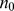
−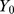).

Regarding phase I, assuming that 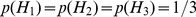, three possibilities could be considered: **(1)** patients in the first cohort are treated at the lowest dose 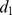; **(2)** at the current dose level 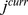 with the observed data 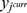, we calculate 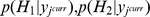 and 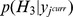. If 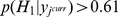, we escalate the dose level to 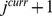; if 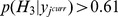, we de-escalate the dose level to 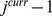; and if 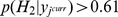, the dose stays at the same level as 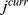 for the next cohort of patients; **(3)** otherwise, we switch to the mTPI method to continue the dose-finding jobs.

During the phase I process, if there is/are promising dose(s) graduated to phase II, the proposed design adaptively randomizes to-be-treated patients to all the graduated doses or placebo. In our design, the cohort size is 3 patients in phase II, the estimated response rates for dose levels 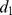 to 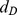 are denoted by 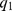 to 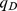, respectively.

Adaptive allocation procedure has the feature of assigning the patients to the most efficacious dose. The mechanism of allocation of patients is the essential part of an adaptive randomization approach. There are several approaches that have been proposed in recent years ([1], [16], [17]). One traditional approach is to let the assignment probability for dose 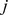 be proportional to its response rate 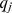 evaluated by the accumulative information so far. This approach does not perform well when the sample size is small. However, small sample size characterizes in every early phase studies. The problem is that the estimated values of 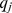 is not reliable and stable. A Bayesian approach is to compare the response rates with a target rate, say 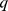, and let the allocation probabilities be proportional to the posterior probabilities 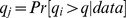. Nonetheless, as pointed out in [15], adaptive randomization may not work well with this approach when all of the true response rates are much higher or lower than 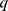. In our design, we adopt the approach proposed by [15], named as Bayesian moving-reference adaptive randomization(MAR) approach. Peculiar to MAR is that the set of treatments in comparison is continuously reduced and one can achieve a high resolution to distinguish various treatments thereby. In the following, we explicate the MAR approach. Firstly, Let 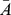 and 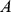 denote the set of indices of the treatment arms that have or have not been assigned randomization probabilities. One starts with 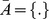 an empty set, and 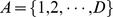;secondly, compute the average response rate for the arms belonging to the set 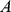, 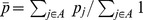, and use 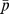 as the reference to determine 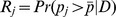, for 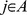. Identify the arm that has the smallest value of 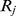, 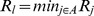; then assign arm 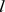 a randomization probability of 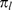, 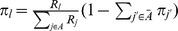, and update 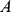 and 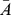 by removing arm 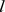 from 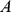 into 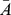; lastly, repeat the first two steps and keep spending the rest of the randomization probability until all of the arms are assigned randomization, 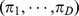, and then randomize the next cohort of patients to the j-th arm with a probability of 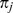. This approach can overcome the disadvantage mentioned above. The detailed description of this approach is refereed to [15].

We propose the following rules that are applied to the accumulating data for each dose arm.

- *Rule1:Futility rule (dose dropping)*: Calculate the posterior probability 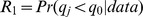, where 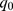 is the placebo arm’s response rate. Stop accruing patients at this dose level j whenever 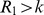.
- *Rule2:Graduation rule(dose expansion)*: If the dose arm’s posterior probability 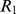 in *Futility rule*, compute 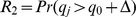, where, 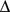 is a physician-specified superiority treatment margin. If 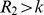, graduate this dose to phase II.

In sum, our proposed design is schematized as follows:

### 

#### Trial initiation

Patients of the first cohort are treated at the lowest dose level.

#### Onset of phase I

Phase I dose-finding starts after the first cohort is enrolled. Dose escalation proceeds based on the hybrid mTPI design.

#### Dose expanding

If an adjacent higher dose arm is opened for safety testing, we simultaneously expand the current administered dose to phase II.

#### Onset of phase II

Once a dose graduates, phase II starts. Patients will be randomized to the graduated doses or a placebo arm. For arm 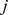, the randomization probability is proportional to the probability computed by the MAR approach.

#### Trial termination

The trial is terminated when either of the two conditions is met: 1) no dose is left in both phases; or 2) the prespecified maximum sample size is reached.

## Simulation Studies

### Simulation settings

For the purpose of fair comparison, we use the simulation scenarios identical to Xie([7]), whose study consists of two sets of toxicity situations: equal toxicity rates, with 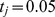 for 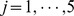, and increasing toxicity rates(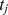
s) with 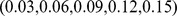 for all dose levels. Two control response rates(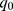) used in the simulation are 0.2 and 0.5. The true treatment response rate(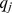) used in simulations intends to encompass the various scenarios occurred in the real practice, namely, null, increasing, decreasing, n-shaped, u-shaped and equal (please refer to Table 1 for details).

**Table 1:** Dose response rates(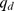) and Placebo response rate(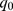) scenarios.

Pattern (*d*_1_, *d*_2_, *d*_3_, *d*_4_, *d*_5_)	Dose Response Rate( 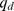 )	Placebo Response Rate( 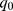 )
Null	0.2,0.2,0.2,0.2,0.2	
Increasing	0.2 0.3 0.5 0.7 0.8	
Decreasing	0.8 0.7 0.5 0.3 0.2	0.2
n-shaped	0.2 0.4 0.8 0.4 0.2	0.5
u-shaped	0.8 0.4 0.2 0.4 0.8	
Equal	0.5 0.5 0.5 0.5 0.5	

In the simulations, the parameters are selected with exploratory simulation studies by computing the competing designs to achieve the similar type I error rate: 0.05. The maximum sample size for the trial is 180 and, the maximum sample size for each dose arm or placebo arm is 30 patients. Δ is 0.2, and the cutoff k is set as 0.90 by calibration. For each of the 24 scenarios, the proposed design was compared to both the XJT design and the three-stage design varied in terms of the average total sample size, optimal dose selection percentage, average patient numbers on various dose levels and toxic rates.

### Simulation results

#### Sample size reduction

When toxicity pattern is either equal or increasing, the average number of patients using the XJT design is 135 or 129 for 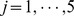. Compared to the XJT design, the average sample size of our proposed design is vastly saved across all scenarios, approximately 25% sample size reduction on average; besides as against the three-stage design, the average sample consumption is also saved dramatically, approximately 12% sample size reduction on average. (See Table 2 for details). As is obvious from the above results, the proposed design is very competitive in terms of cost, with the implication of shorter drug development duration and higher ethics due to smaller sample size. The good performance is traceable to the fact that our proposed design adopts the efficient hybrid mTPI design in phase I and an adaptive randomization procedure in phase II.

**Table 2:** Average Total Sample Size and Percentage Reduction.

	Equal Toxicity						Increasing Toxicity					
	(0.05,0.05,0.05,0.05,0.05,0.05)						(0.03,0.06,0.09,0.12,0.15)					
Placebo rate	Null	Increasing	Decreasing	n-shaped	u-shaped	Equal	Null	Increasing	Decreasing	n-shaped	u-shaped	Equal
0.2	101	69	67	67	65	75	80	79	67	63	65	64
	(4)	(47)	(51)	(46)	(57)	(60)	(9)	(37)	(51)	(45)	(60)	(44)
	(2)	(26)	(26)	(20)	(26)	(25)	(2)	(18)	(32)	(33)	(31)	(23)
0.5	97	102	99	96	100	84	84	100	109	96	105	123
	(13)	(11)	(10)	(13)	(8)	(22)	(18)	(18)	(7)	(18)	(9)	(11)
	(6)	(5)	(4)	(5)	(2)	(11)	(8)	(7)	(2)	(8)	(3)	(4)

Note: The first row is average total sample size in our proposed design; numbers in parentheses are percentages reduction of sample size as compared to the XJT and the three-stage designs.

#### Optimal allocations

The dose selection percentages and average numbers of patients treated upon various doses are presented in Table 3. With toxicity and efficacy increasing, scenarios 14 and 20 are the most encountered situations in clinical practice. It is easy to see, in two cases, the dose selection percentages for doses 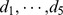 are increasing from 13.7 to 28.9 and from 12.4 to 25.6 respectively, and the average number of patients assigned to the various doses is increasing from 8.3 to 15.1 and from 11.1 to 23.1 correspondingly. To examine the net effect of the number of patients assigned to an increasing dose response, for instance, in scenario 2, the toxic rates are constant across the various doses, while the response rates exhibit an increasing trend from 20% to 60%. As shown in Table 3, the number of patients assigned to various doses ranges from 7.9 to 19.3, or 11.4 to 34.2 in percentage. The above results show that the proposed design efficiently assigns more patients to the most effective dose levels. The Scenario 8 shows the similar results. When it comes to the net effect of increasing toxic rates as in scenario 13, the response rates remain unchanged, while the toxic rates vary between 3% and 15%. From Table 3, the average sample size consumed by doses decreases from 17.3 to 9.9, or from 26.4 to 14.1 in terms of percentage. The Scenario 19 also exhibits similar results.

**Table 3:** Dose selection percentage and average number patients allocated to each dose under proposed design.

		Equal Toxicity						Increasing Toxicity				
Sc.	Control resp rate	(0.05,0.05,0.05,0.05,0.05)					Sc.	(0.03,0.06,0.09,0.12,0.15)				
1	0.2	0.2	0.2	0.2	0.2	0.2	13	0.2	0.2	0.2	0.2	0.2
	%	(21.3)	(20.8)	(19.7)	(20.1)	(20.5)	%	(**26.4**)	(22.1)	(19.9)	(18.1)	(14.1)
	#	(19.2)	(18.6)	(18.1)	(17.3)	(18.1)	#	(**17.3**)	(15.5)	(14.1)	(12.8)	(9.9)
2	0.2	0.2	0.3	0.4	0.5	0.6	14	0.2	0.3	0.4	0.5	0.6
	%	(11.4)	(13.2)	(14.9)	(26.1)	(**34.2**)	%	(13.7)	(16.2)	(20.1)	(24.6)	(**28.9**)
	#	(7.9)	(9.1)	(12.2)	(13.9)	(**19.3**)	#	(8.3)	(10.5)	(12.3)	(14.2)	(**15.1**)
3	0.2	0.6	0.5	0.4	0.3	0.2	15	0.6	0.5	0.4	0.3	0.2
	%	(**39.4**)	(25.9)	(16.9)	(12.5)	(11.1)	%	(**38.2**)	(24.9)	(15.7)	(13.1)	(8.8)
	#	(**21.2**)	(15.3)	(10.7)	(8.3)	(5.1)	#	(**20.9**)	(14.8)	(9.9)	(7.9)	(5.9)
4	0.2	0.2	0.4	0.6	0.4	0.2	16	0.2	0.4	0.6	0.4	0.2
	%	(11.4)	(18.9)	(**44.1**)	(13.6)	(8.7)	%	(12.5)	(23.1)	(**43.9**)	(14.6)	(5.9)
	#	(8.1)	(11.6)	(**23.3**)	(10.0)	(5.6)	#	(8.2)	(11.5)	(**21.7**)	(9.1)	(5.3)
5	0.2	0.6	0.4	0.2	0.4	0.6	17	0.6	0.4	0.2	0.4	0.6
	%	(**36.3**)	(16.2)	(9.1)	(12.2)	(**29.7**)	%	(**37.9**)	(15.1)	(10.0)	(11.3)	(24.7)
	#	(**19.9**)	(9.2)	(6.8)	(8.1)	(**15.6**)	#	(**19.7**)	(9.9)	(6.5)	(8.0)	(13.6)
6	0.2	0.5	0.5	0.5	0.5	0.5	18	0.5	0.5	0.5	0.5	0.5
	%	(21.8)	(20.5)	(20.1)	(19.3)	(18.3)	%	(**24.7**)	(25.9)	(23.7)	(19.2)	(10.2)
	#	(14.8)	(14.5)	(13.1)	(13.9)	(14.0)	#	(**15.5**)	(12.5)	(11.9)	(10.1)	(7.4)
7	0.5	0.5	0.5	0.5	0.5	0.5	19	0.5	0.5	0.5	0.5	0.5
	%	(21.2)	(20.4)	(19.7)	(18.3)	(17.6)	%	(**25.1**)	(23.2)	(21.0)	(17.4)	(14.1)
	#	(17.1)	(16.4)	(16.1)	(15.7)	(14.8)	#	(**18.6**)	(16.9)	(14.6)	(12.2)	(10.8)
8	0.5	0.5	0.6	0.7	0.8	0.9	20	0.5	0.6	0.7	0.8	0.9
	%	(11.6)	(14.3)	(18.5)	(24.1)	(**28.7**)	%	(12.4)	(15.3)	(21.5)	(22.8)	(**25.6**)
	#	(10.5)	(14.3)	(19.8)	(22.6)	(**26.1**)	#	(11.1)	(13.7)	(19.4)	(21.6)	(**23.1**)
9	0.5	0.9	0.8	0.7	0.6	0.5	21	0.9	0.8	0.7	0.6	0.5
	%	(**39.6**)	(25.3)	(15.1)	(10.0)	(8.3)	%	(**38.8**)	(27.8)	(17.2)	(10.1)	(6.2)
	#	(**28.6**)	(24.4)	(18.0)	(13.1)	(7.9)	#	(**29.0**)	(25.1)	(18.9)	(12.7)	(8.1)
10	0.5	0.5	0.7	0.9	0.7	0.5	22	0.5	0.7	0.9	0.7	0.5
	%	(13.1)	(22.8)	(**39.7**)	(18.1)	(8.1)	%	(11.5)	(25.6)	(**40.1**)	(17.7)	(7.4)
	#	(11.3)	(21.8)	(**27.4**)	(18.9)	(8.8)	#	(10.5)	(21.6)	(**28.9**)	(19.2)	(7.6)
11	0.5	0.9	0.7	0.5	0.7	0.9	23	0.9	0.7	0.5	0.7	0.9
	%	(**39.2**)	(18.3)	(7.1)	(13.2)	(**24.4**)	%	(**38.5**)	(22.1)	(9.4)	(13.4)	(21.1)
	#	(**29.1**)	(19.7)	(8.9)	(18.6)	(**23.2**)	#	(**29.4**)	(21.1)	(7.3)	(16.2)	(19.7)
12	0.5	0.8	0.8	0.8	0.8	0.8	24	0.8	0.8	0.8	0.8	0.8
	%	(20.8)	(20.7)	(19.4)	(19.7)	(19.1)	%	(**28.3**)	(24.5)	(21.7)	(15.2)	(12.7)
	#	(14.1)	(14.0)	(13.7)	(13.9)	(13.7)	#	(**27.8**)	(25.2)	(24.8)	(20.5)	(16.9)

Note: In each scenario, the first row of number in parentheses corresponds to the dose selection percentage at every dose combination, the second corresponds to the average number of patients allocated to every dose combination.

In brief, the proposed design saves a lot sample size and, in all scenarios, the optimal doses are to be selected with high probability and a large proportion of patients can be assigned to the efficacious and safe dose levels.

## Discussion

Early phases in clinical trials, like phase I and II, play a vital role in drug development. The success of phase III confirmatory trials is contingent on phase I and II. However, the traditional procedures separate the early phases into two distinct phases and fail to borrow the information across phases I and II. Therefore, a design that could efficiently integrate information accumulated in phases I and II would be especially beneficial and necessary to drug development, with reference to the current situation of high risk and cost. The design described in this paper intends to provide an upgraded version based on the three-stage design. The highlights of our proposed design in this paper embrace adoption of a novel phase I bayesian design - the hybrid mTPI design and the MAR randomization procedure. Extensive simulation studies are conducted to ascertain the claimed good performances.

Readers may wonder why we did not choose a hybrid CRM in phase I stage. Basically, the CRM approach requires one to select a group of skeletons prior to a trial, but how to choose the skeletons remains an unsolved academic problem, despite that one method has been proposed ([13]). In fact, we have done the study using the hybrid CRM in phase I stage, yet not present them in tables. The findings, actually, lead to the same results as the hybrid mTPI. Accordingly, we believe that the hybrid mTPI approach would be much easier and more user-friendly to be adopted in real practice.

In the final analysis, the design proposed in the paper is more ethical with more patients assigned to the optimal doses, can expedite the clinical procedures, and can save the cost of drug development due to small sample size. The design structure is easy to understand and practice of this design is free from calibration of design parameters.
