# Correction: A Novel Bayesian Seamless Phase I/II Design

**DOI:** 10.1371/annotation/fff74def-44d5-4eab-9d92-39fae778bd36

**Published:** 2013-10-01

**Authors:** Haitao Pan, Ping Huang, Zuoren Wang, Ling Wang, Chanjuan Li, Jielai Xia

There was an error in the Funding statement. The correct version of the Funding statement is available below.

This work was partially supported by Research Grant 81273176 and 81001290 from National Science Foundation of China. The first author’s work was partially supported by the Scientific Research Project of Education Department of Shaanxi Province (Grant 2013JK0762) and the National Science Foundation of China (81302513). No other additional external funding was received for this study. The funders had no role in study design, data collection and analysis, decision to publish, or preparation of the manuscript. 

